# Laser and energy‐based devices for treating rosacea ‐ a systematic review and network meta‐analysis

**DOI:** 10.1111/ddg.15961

**Published:** 2025-11-21

**Authors:** Lynhda Nguyen, Christina Sorbe, Nikolaus Seeber, Stefan W. Schneider, Katharina Herberger

**Affiliations:** ^1^ Department of Laser and Aesthetics Department for Dermatology and Venereology University Medical‐Center Hamburg‐Eppendorf Hamburg Germany; ^2^ Institute for Health Services Research in Dermatology and Nursing University Medical Center Hamburg‐Eppendorf Hamburg Germany; ^3^ Joint Practice for Dermatology Dres. Peter/Seeber/Altheide Hamburg Germany; ^4^ Department for Dermatology and Venereology University Medical‐Center Hamburg‐Eppendorf Hamburg Germany

**Keywords:** Energy‐based devices, IPL, laser, network meta‐analysis, rosacea

## Abstract

**Background and Objectives:**

Rosacea is a chronic inflammatory condition affecting the central face. While laser and energy‐based devices (EBDs) are commonly used for managing vascular symptoms, comprehensive comparisons between systems are limited. This study aims to evaluate and compare the efficacy and safety of lasers and EBDs for rosacea through a systematic review and network meta‐analysis (NMA).

**Methods:**

Randomized controlled trials (RCTs) were identified from MEDLINE, CENTRAL, and Web of Science, with additional searches for ongoing trials. Primary outcomes included patient satisfaction, improvements in erythema and telangiectasia, and long‐term adverse events (AEs). Risk of bias (RoB) and publication bias were also assessed.

**Results:**

25 RCTs were included for qualitative analysis, and subsets contributed to the NMAs. Most studies exhibited unclear or high risk of bias, with a slight publication bias observed. Radiofrequency microneedling was more effective than pulsed dye laser (PDL) in terms of patient satisfaction (MD –1.32; 95 % CI –1.89 to –0.76) and erythema (MD –1.44; 95 % CI –1.96 to –0.91). The combination of oxymetazoline and PDL appeared superior for telangiectasia (MD –0.58; 95 % CI –1.03 to –0.14). AEs and discontinuation rates were comparable across treatments.

## INTRODUCTION

Rosacea is a chronic inflammatory skin disease primarily affecting the central areas of the face. The reported prevalence of rosacea varies significantly across studies, ranging from less than 1 % to 22 %.^1^ The disease manifests in a wide range of phenotypes, from transient and persistent erythema to telangiectasia, inflammatory lesions, and phymatous changes.^2^ In addition to these primary symptoms, patients may experience discomfort like itching or burning. The striking appearance of rosacea can also affect self‐esteem and have a negative effect on patients' quality of life.^3^

A variety of treatment options are available for managing rosacea, including topical and systemic medications, laser‐ and energy‐based devices (EBDs), and combination therapies, particularly in more severe cases.^4^ In recent years, laser and energy‐based therapies have been established modalities to treat rosacea.^4^ These treatments work on the principle of selective photothermolysis, where specific wavelengths are absorbed by the targeted chromophores only.^5^ In rosacea, hemoglobin and oxyhemoglobin serve as the key chromophores, with hemoglobin showing absorption peaks at 432 nm and 556 nm, while oxyhemoglobin absorbs at 414 nm, 542 nm, and 576 nm.^6^

Among lasers and EBDs, pulsed dye lasers (PDL) are currently regarded as the treatment of choice for treating rosacea. Given the wide range of systems now available for managing vascular‐based conditions, a thorough comparison is necessary. Conventional meta‐analyses are limited in their capacity to assess multiple treatments simultaneously. However, a network meta‐analysis (NMA), which enables both direct and indirect comparisons, offers a solution to this limitation.

To the best of our knowledge, no studies have yet established a comparative network of laser and energy‐based treatments for rosacea. Therefore, this study aims to systematically evaluate the efficacy, safety, and tolerability of all available laser and energy‐based treatments for rosacea.

## MATERIAL AND METHODS

### Protocol and registration

A systematic review and NMA of randomized controlled trials (RCTs) of laser‐ and energy‐based treatments for rosacea was performed according to the extended *Preferred Reporting Items for Systematic Reviews and Meta‐Analysis* (PRISMA) guidelines.^7^, ^8^ According to the PICO framework, the *population* were rosacea patients. The *intervention* and the *comparator* were laser and energy‐based treatments. The *outcome* was patient satisfaction, investigator‐assessed reduction in erythema and telangiectasia, and long‐ and short‐term adverse events (AEs). Study selection, data extraction, and risk of bias (RoB) assessments were conducted independently by two authors (L.N., K.H.). In case of discrepancies, a third reviewer (S.W.S.) was consulted. Study protocol was preregistered on PROSPERO (CRD42023485884).

### Search strategy

We searched following databases up until 23 November 2023: MEDLINE (Ovid), Cochrane Central Register of Controlled Trials (CENTRAL), and Web of Science. In addition, the trial registers WHO Trials Registry and Clinicaltrials.gov were searched up to 25 December 2023. There was no language restrictions applied to the search. Detailed search strategy can be found in online supplementary material S1. Furthermore, a forward (cited‐by) and backwards (reference) search were performed on all included studies using Google Scholar and Web of Science. All identified studies were screened by title and abstract.

### Selection criteria

Full‐article RCTs examining all types of laser‐ or energy‐based interventions for treating rosacea were included. Studies focusing on facial telangiectasias were also eligible for inclusion. Those focusing on rhinophyma, ocular rosacea, or Morbus Morbihan were excluded, as these represent distinct subtypes of rosacea.^9^ Trials that assessed only a single intervention were considered as one‐arm studies and were therefore not included in the quantitative analysis.

### Data extraction

Following data were extracted: First author's name, publication year, number of participants, sex and age of participants, Fitzpatrick skin types, phenotypes of rosacea, interventions, outcomes, short‐ and long‐term AEs, and withdrawals due to AEs.

### Primary and secondary outcomes

The primary outcomes assessed were patient satisfaction, improvements in erythema and telangiectasia, and long‐term AEs such as post‐inflammatory hyperpigmentation and scarring, which can negatively affect the quality of life. Additionally, withdrawals due to AEs were considered. Secondary outcomes included short‐term AEs like crusts, swelling, and erythema. Notably, purpura was not considered an AE, as it is the clinical endpoint of several laser treatments.

### Risk of bias assessment

The RoB was assessed using the Cochrane RoB tool for randomized trials (RoB 2).^10^ This tool encompasses following domains: *(1)* random sequence generation, *(2)* allocation concealment, *(3)* blinding of participants and personnel, *(4)* blinding of outcome assessment, *(5)* incomplete outcome data, *(6)* selective reporting, and *(7)* other bias. Each domain can be classified as low risk, high risk, or unclear risk of bias.^10^

### Publication bias

Publication bias was assessed through a funnel plot, Egger's test, and a non‐parametric trim‐and‐fill approach using the metafor package in R.^11^, ^12^

### Statistical analysis

Data analysis was conducted using Review Manager 5 (Cochrane Collaboration). A meta‐analysis assessed lesion clearance efficacy compared to 595 nm PDL, using the mean differences (MD) with 95 % confidence intervals (CI) and random‐effects models. Median and interquartile ranges, commonly reported in the studies, were converted to arithmetic means and standard deviations using the Box‐Cox method.^13^, ^14^ Statistical calculations were performed in R (version 4.3.1) with the netmeta package (version 2.8.2) and the estmeansd package (version 1.0.0).^13^, ^14^, ^15^, ^16^ An additive NMA model was employed to evaluate treatment effects and measure inconsistency (I^2^) within the NMA framework.^17^ p values < 0.05 were considered significant.

## RESULTS

### Search results

**FIGURE 1:**
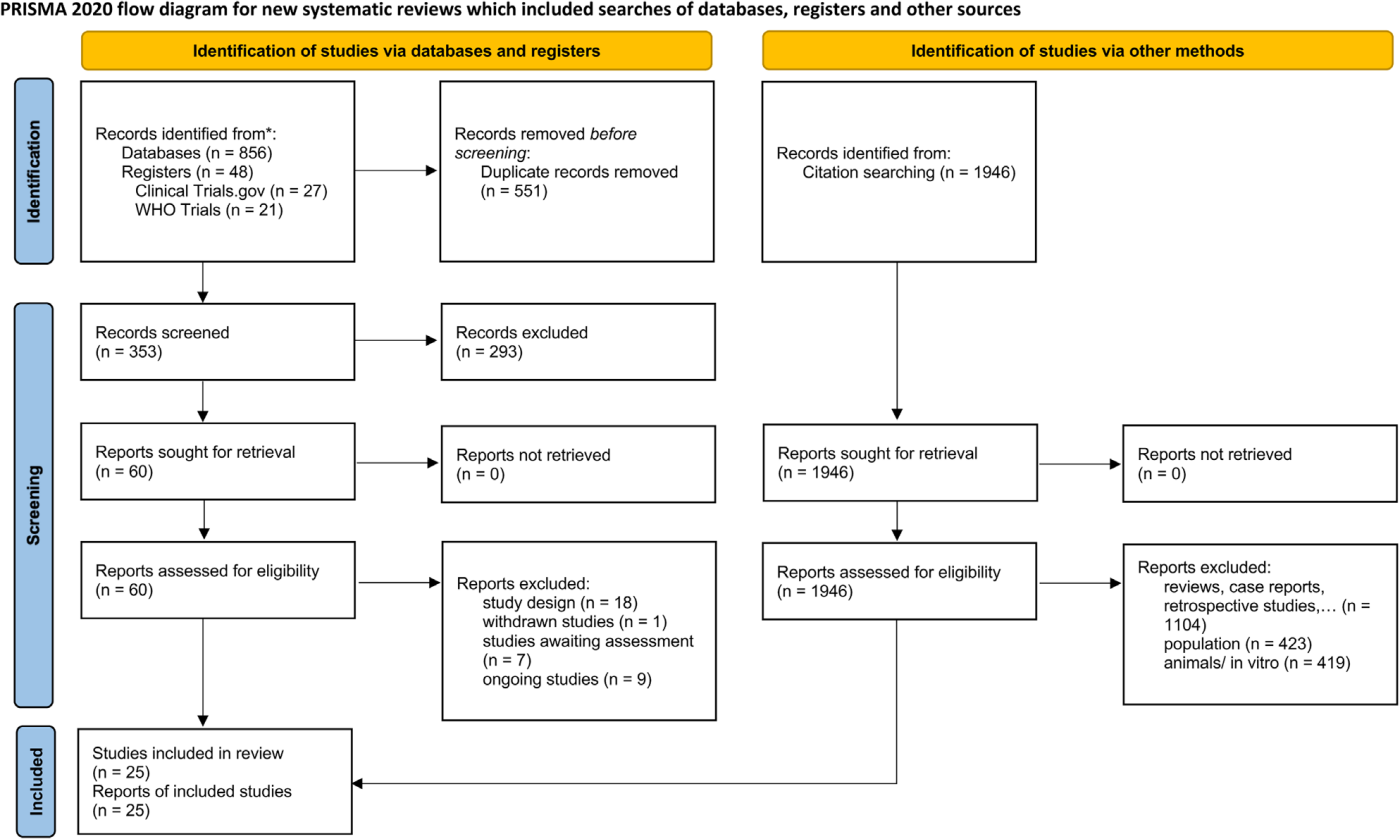
Study flowchart.

The initial systematic literature search identified 856 publications and 48 trial registrations. A forward and backward search uncovered an additional 1,946 potential studies. After removing duplicates and screening potential records by title and abstract, 60 articles remained for further eligibility assessment. After additional screening, 25 studies were included in the qualitative analysis.^18^, ^19^, ^20^, ^21^, ^22^, ^23^, ^24^, ^25^, ^26^, ^27^, ^28^, ^29^, ^30^, ^31^, ^32^, ^33^, ^34^, ^35^, ^36^, ^37^, ^38^, ^39^, ^40^, ^41^, ^42^ Sixteen were included in the quantitative analysis (Figure 1).^18^, ^19^, ^20^, ^22^, ^24^, ^25^, ^26^, ^28^, ^31^, ^32^, ^33^, ^34^, ^35^, ^36^, ^38^, ^42^ Detailed information regarding all included and excluded studies can be found in online supplementary material S2 and S3.

### Trial characteristics

The 25 RCTs included in the qualitative analysis comprised a total of 843 patients, with a mean age of 46.9 years (18–85). Gender data was missing for 48 patients.^39^, ^42^ Among the reported demographics, 66.3 % were female. Fitzpatrick skin type was unreported in 14 publications.^24^, ^25^, ^27^, ^31^, ^35^, ^36^, ^38^, ^39^, ^40^ Of the patients in the remaining studies, the distribution was as follows: 8.3 % had Fitzpatrick skin type I, 36.6 % had Fitzpatrick skin type II, 32.2 % had Fitzpatrick skin type III, 20 % had Fitzpatrick skin type IV, and 2.9 % had Fitzpatrick skin type V. No patients with Fitzpatrick skin type VI were included. Of the included studies, 76 % reported on treatments for persistent erythema encompassing 690 participants,^18^, ^19^, ^20^, ^21^, ^24^, ^25^, ^26^, ^27^, ^28^, ^29^, ^30^, ^31^, ^32^, ^33^, ^34^, ^37^, ^38^, ^40^, ^41^ and 88 % addressed the treatment of telangiectasia involving a total of 784 participants.^18^, ^19^, ^20^, ^21^, ^22^, ^23^, ^25^, ^26^, ^27^, ^28^, ^29^, ^30^, ^31^, ^32^, ^33^, ^34^, ^35^, ^36^, ^38^, ^39^, ^40^, ^41^, ^42^ For full details, see online supplementary material S2.

### Quality Assessment

**FIGURE 2:**
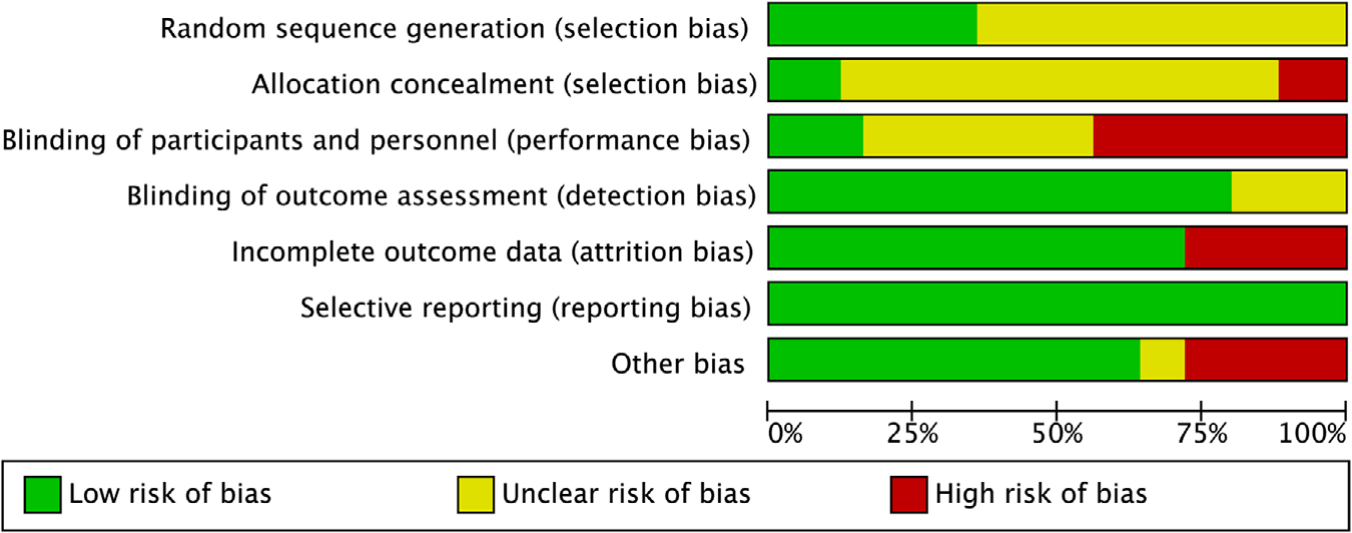
Risk of bias graph: summary of the authors’ assessment of each risk‐of‐bias item, shown as percentages across all included studies. Green (+): low risk of bias; yellow (±): unclear risk of bias; red (–): high risk of bias.

**FIGURE 3:**
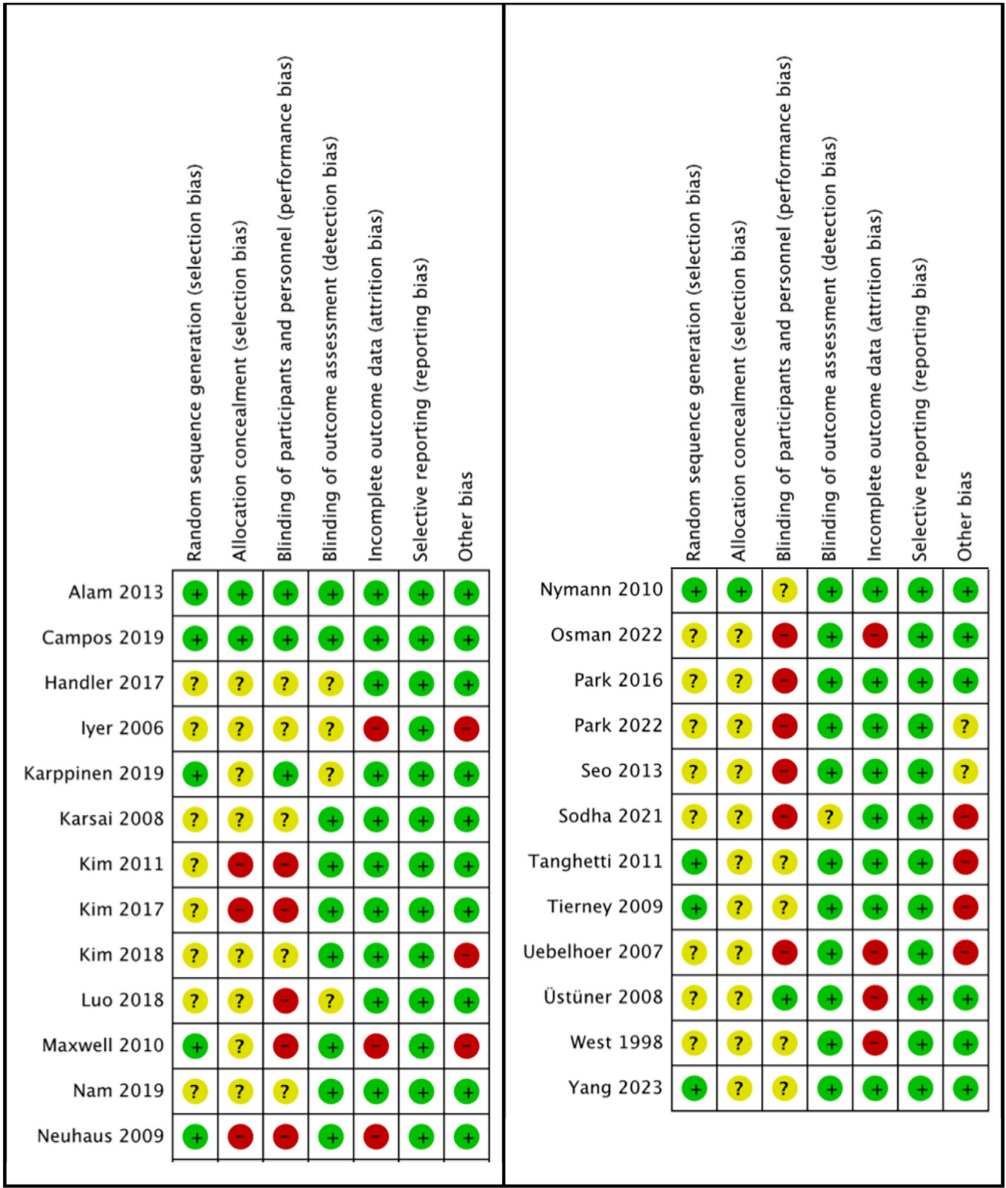
Summary of the authors’ evaluations of each risk‐of‐bias item for each included study.

Most studies exhibited an unclear RoB in at least one domain, primarily due to inadequate reporting (Figures 2, 3). The risk associated with performance blinding and selection bias was often unclear or high, mostly owing to the nature of device‐based treatments. Detailed analyses of the RoB for each included study can be found in online supplementary material S2.

For patient satisfaction, funnel plot analysis showed a uniform outcome distribution with one outlier (RFMN). Egger's test (p < 0.1925) indicated no publication bias. The trim‐and‐fill method added no studies. For erythema, the funnel plot showed an even distribution with one observation (RFMN) outside the expected range. Egger's test (p < 0.150) suggested no significant bias, and the trim‐and‐fill method added one study, indicating minimal publication bias. For telangiectasia, the funnel plot showed two outliers (IPL; niacin + PDL) with a symmetrical distribution otherwise. Egger's test (p < 0.149) and the addition of three studies by the trim‐and‐fill method indicate moderate publication bias. For details see online supplementary material S4.

### Treatment results

**FIGURE 4:**
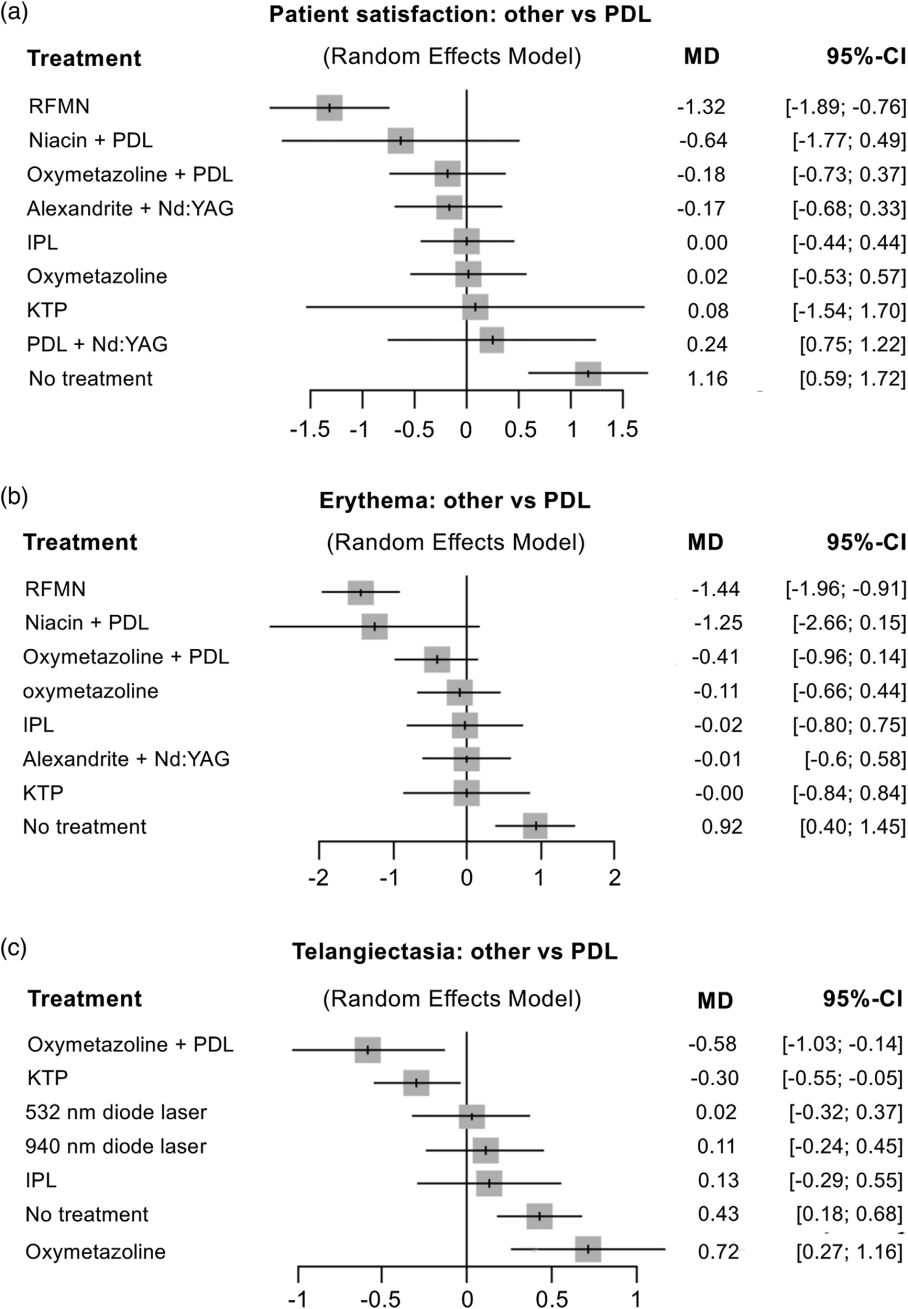
Network meta‐analysis of (a) patient satisfaction, (b) efficacy in treating erythema, and (c) telangiectasia in rosacea with various laser and energy‐based treatments compared to pulsed‐dye laser (PDL). A mean difference (MD) of < 0 indicates a greater likelihood of preference for the comparator treatments. Abbr.: MD, mean difference; CI, confidence interval; RFMN, radiofrequency microneedling; IPL, intense pulsed light; Nd:YAG, neodymium‐doped yttrium aluminum garnet; KTP, potassium titanyl phosphate

To address the variability in assessment methods across the included studies, data were standardized to a visual analog scale ranging from 1 to 5 (clear to severe), which was the most used method. The patient satisfaction and efficacy of lasers and EBDs in treating the erythema and telangiectasia component, compared to PDL monotherapy, are illustrated in the forest plots (Figure 4). Treatments are ranked from most to least effective.

#### Patient satisfaction

For patient satisfaction, seven studies contributed to the quantitative efficacy analysis. The NMA included seven pairwise comparisons across ten different treatment approaches, comprising 321 observations and three sub‐networks (Figure 4, online supplementary material S5). RFMN (MD –1.32; 95 % CI –1.89 to –0.76) was found to be significantly more effective in reducing erythema in rosacea, followed by niacin + PDL (MD –0.64; 95 % CI –1.77 to 0.49) and oxymetazoline + PDL (MD –0.18; 95 % CI –0.73 to 0.37). The least promising approach was no treatment (MD 1.16; 95 % CI 0.59 to 1.72).

#### Erythema

A total of eight studies contributed to the quantitative analysis of erythema improvement. The NMA included eight pairwise comparisons involving ninbe different treatment approaches, with 272 observations and three sub‐networks (Figure 4, online supplementary material S5). RFMN (MD –1.44; 95 % CI –1.96 to –0.91) demonstrated significant efficacy in treating erythema in rosacea. In contrast, no treatment was identified as the least effective method (MD 0.92; 95 % CI 0.40 to 1.45).

#### Telangiectasia

In the NMA for telangiectasia, five studies were included in the quantitative efficacy analysis. This NMA comprised five pairwise comparisons across eight treatment approaches, with 177 observations and four sub‐networks (Figure 4, online supplementary material S5). Oxymetazoline + PDL (MD ‐0.58; 95 % CI –1.03 to –0.14) was shown to be significantly more effective in treating telangiectasia in rosacea, followed by KTP (MD –0.30; 95 % CI –0.55 to –0.05). The least effective treatments were oxymetazoline (MD 0.72; 95 % CI 0.27 to 1.16) and no treatment (MD 0.43; 95 % CI 0.18 to 0.68).

#### Adverse events

All studies included in the analysis reported AEs, but only six provided data on the prevalence of short‐term AEs, which included post‐treatment erythema, swelling, crusting, and blistering, all of which resolved within a few days.^19^, ^20^, ^24^, ^26^, ^32^, ^36^ Long‐term AEs were documented in 15 studies.^20^, ^22^, ^24^, ^26^, ^27^, ^28^, ^29^, ^31^, ^32^, ^33^, ^34^, ^35^, ^36^, ^40^, ^41^ Further details can be found in online supplementary material S2.

Due to incomplete data, a meta‐analysis on AEs could not be performed. Karppinen et al. (2019) found superficial atrophic scars in 11.1 % of patients treated with the 585 nm yellow laser.^22^ Luo et al. (2020) observed long‐term AEs in 10.8 % of cases after IPL treatment, including facial burning, hyperpigmentation, and blisters.^27^ Additionally, Seo et al. (2013) documented post‐treatment hyperpigmentation in one patient out of 18 treated with IPL and 1 out of 19 treated with PDL.^33^

#### Discontinuation due to adverse events

No meta‐analysis was performed due to the lack of significant differences between treatments. Alam et al. (2013) reported that two out of 16 patients withdrew because of post‐treatment swelling, though it was unclear if this was after PDL or Nd:YAG treatment.^18^ Similarly, Campos et al. (2019) noted two out of 29 discontinuations, one for unacceptable purpura and another for excessive pain, without specifying the responsible laser.^19^ Seo et al. (2016) observed that one out of 25 patients treated with PDL withdrew due to worsening symptoms.^33^ Üstüner et al. (2018) reported two out of 30 dropouts from either KTP or Nd:YAG lasers due to AEs.^41^

In Luo et al. (2020), four out of 130 control patients who received no treatment experienced rosacea aggravation, while six out of 130 IPL patients dropped out due to blisters, burning, redness, or edema.^27^ Neuhaus et al. also noted that one out of 30 IPL‐treated patients withdrew due to excessive swelling, although this reaction could not be further evaluated due to loss of follow‐up.^29^

Additionally, one patient (1/18) treated with a combination of PDL and oxymetazoline withdrew for unspecified reasons, while another (1/16) receiving only topical oxymetazoline also dropped out.^34^ Lastly, Yang et al. (2023) reported one dropout among 22 patients taking minocycline (100 mg) due to AEs.^40^

## DISCUSSION

This systematic review with NMA offers a comparative analysis of the efficacy, safety, and tolerability of various laser‐ and energy‐based treatments for rosacea, based on the available RCTs published to date.

Unlike other systemic and topical pharmaceutical treatments, our analysis indicates that the evidence supporting the use of lasers and EBDs for rosacea remains relatively uncertain. Consistently, recent reviews suggest low‐to‐moderate‐certainty evidence that PDL, Nd:YAG laser, and IPL are effective in reducing background erythema and telangiectasia in rosacea patients.^4^, ^43^ This NMA also included unconventional treatment approaches for rosacea, such as the combination of oxymetazoline and PDL, as well as RFMN therapy. These methods demonstrated promising results in improving patient satisfaction and reducing rosacea symptoms. However, funnel‐plot analysis identified RFMN as an outlier in these categories, indicating a potential publication bias. In particular, the combination of PDL and topical oxymetazoline showed the highest relative efficacy in our analysis for treating rosacea‐associated telangiectasia. However, this ranking is based on available RCT data and may not reflect broader clinical experience or long‐term outcomes. KTP lasers have long been established as highly effective and well‐tolerated in routine practice, with high clearance rates and strong patient satisfaction.^44^

The findings of the present study must be interpreted cautiously, as they are supported by a limited number of studies with small sample sizes. Furthermore, many of the included studies exhibited a high RoB, primarily due to insufficient reporting of blinding and randomization processes. Additionally, laser and energy‐based devices, even those operating at the same wavelength, can vary significantly and therefore, substantially influence both treatment efficacy and safety profiles.

Combination therapies are often recommended for managing more severe cases of rosacea.^9^ In our analysis, we included all studies that had at least one treatment arm involving a laser or EBD. However, there were only few studies focusing on combination therapies. Tailoring treatments to the specific clinical phenotype of rosacea is essential for achieving optimal therapeutic outcomes. Combination approaches – whether utilizing multiple devices or pairing laser or EBDs with topical or systemic agents – have shown potential for improving patient outcomes, especially in addressing the multifaceted nature of rosacea symptoms.^45^ Further investigation is warranted to fully explore the efficacy and safety of these combined approaches.

Patients and physicians need to align on the expected outcomes, especially as laser and EBD therapies typically provide temporary relief and often require multiple sessions for sustained results. Patients should be fully informed about the treatment process, including the expected course of treatment, the healing process, the outcomes, and any potential risks or complications specific to the laser or EBD treatment being used.

The included studies predominantly involved female patients. However, epidemiological studies show an equal gender distribution, suggesting that the higher female representation in treatment studies could be due to differences in healthcare‐seeking behavior rather than actual disease prevalence.^46^

Regarding skin types, less than 3 % of the included patients had Fitzpatrick skin type V, and no patients had Fitzpatrick skin type VI. This aligns with the notion that rosacea is less frequently diagnosed in individuals with skin of color and the higher risk for AEs in laser treatments.^47^ Rosacea symptoms, such as flushing or erythema, may not be as noticeable on darker skin, which could contribute to this issue.^48^ However, a systematic review found no differences in rosacea prevalence based on geographic or ethnic distribution, further emphasizing that the disparity in diagnosis is likely due to under‐recognition rather than lower prevalence.^1^, ^49^ Nevertheless, it has to be kept in mind that most laser‐ and energy‐based treatments used for treating rosacea are less suitable for darker skin types as the absorption of melanin in these types may be too high.

This study has some limitations. The included trials did not evaluate the influence of skin type, age, or rosacea severity, which could significantly affect clinical outcomes. Additionally, the relatively small sample size prevented more detailed sub‐analyses. Furthermore, long‐term follow‐up data are lacking, making it challenging to evaluate the durability of treatment effects or to determine recurrence rates over time. We focused solely on RCTs, which may exclude valuable insights from non‐randomized studies, but this choice was made to ensure the highest quality of evidence.

In conclusion, this systematic review and NMA offers a thorough evaluation of laser and EBDs in the treatment of rosacea, based on existing RCTs. However, data should be interpreted with caution due to the generally unclear to high RoB across most of the studies. To strengthen the evidence, further well‐designed studies are needed to better define the efficacy and safety of each treatment. Additionally, data on the long‐term effects of laser and energy‐based therapies on disease progression remain limited. As with other chronic conditions, the primary goal of treatment extends beyond achieving a cure to include effective symptom, thereby positively influencing the disease's overall course. Future research should focus on optimizing treatment parameters, intervals, patient‐specific factors, and combination therapies to enable more personalized and effective therapeutic approaches.

## FUNDING

This study was supported by the German Society for Dermatological Laser Medicine (Deutsche Dermatologische Lasergesellschaft, DDL).

## CONFLICT OF INTEREST STATEMENT

L.N. and K.H. have received lecture fees from Cynosure Lutronic^®^. All other authors declare no conflict of interest.

## Supporting information



Supplementary information

Supplementary information

Supplementary information

Supplementary information

Supplementary information
