# Supplementary material for: Laser and energy‐based devices for treating rosacea ‐ a systematic review and network meta‐analysis
Source: J Dtsch Dermatol Ges. 2025 Nov 21;24(1):24–32. doi: 10.1111/ddg.15961 (PMC12800891; doi:10.1111/ddg.15961)
Supplement: Supplementary file 1 — Supplementary information [file DDG-24-24-s002.docx]

**Laser- and Light-Based Treatments for Rosacea – a Systematic Review and Network Meta-Analysis**

**MEDLINE**

Ovid MEDLINE(R) and Epub Ahead of Print, In-Process, In-Data-Review & Other Non-Indexed Citations, Daily and Versions <1946 to November 22, 2023>

1 exp Rosacea/ 3615

2 rosacea.ti,ab. 3984

3 (facial and (erythema or redness or telangiectasia or telangiectatic or edema or oedema or "edema*" or "oedema*")).mp. [mp=title, book title, abstract, original title, name of substance word, subject heading word, floating sub-heading word, keyword heading word, organism supplementary concept word, protocol supplementary concept word, rare disease supplementary concept word, unique identifier, synonyms, population supplementary concept word, anatomy supplementary concept word] 6702

4 1 or 2 or 3 11169

5 Lasers/ 41341

6 photodynamic therapy.mp. or Photochemotherapy/ 36154

7 flash lamp.mp. 315

8 Intense Pulsed Light Therapy/ 267

9 radiofrequency.mp. 43387

10 5 or 6 or 7 or 8 or 9 120211

11 4 and 10 285

**Web of Science**

**TI=rosacea**

**AB=rosacea**

TI=acial AND (erythema OR redness OR telangiectasia OR telangiectatic OR edema OR oedema OR "edema*" OR "oedema*")

AB=acial AND (erythema OR redness OR telangiectasia OR telangiectatic OR edema OR oedema OR "edema*" OR "oedema*")

**TI=**(laser OR photodynamic therapy OR flash lamp OR intense pulsed light OR radiofrequency)

**AB=**(laser OR photodynamic therapy OR flash lamp OR radiofrequency)

**Cochrane Central Register of Controlled Trials in The Cochrane Library (CENTRAL)**

**Concept 1: Rosacea**

1# ti, ab: rosacea

2# facial AND (erythema OR redness OR telangiectasia OR telangiectatic OR edema OR oedema OR "edema*" OR "oedema*")

**Concept 2: laser**

3# ti, ab: laser OR photodynamic therapy OR flash lamp OR intense pulsed light OR radiofrequency)

4# 1# OR 2#

5# 3# AND 4#

**WHO Trials Registry**

rosacea AND (laser OR photodynamic therapy OR flash lamp OR radiofrequency)

**Clinical Trials.gov**

rosacea AND (laser OR photodynamic therapy OR flash lamp OR radiofrequency)
