# Supplementary material for: Laser and energy‐based devices for treating rosacea ‐ a systematic review and network meta‐analysis
Source: J Dtsch Dermatol Ges. 2025 Nov 21;24(1):24–32. doi: 10.1111/ddg.15961 (PMC12800891; doi:10.1111/ddg.15961)
Supplement: Supplementary file 2 — Supplementary information [file DDG-24-24-s004.docx]

**Characteristics of included studies [ordered by study ID]**

| **Alam 2013** | | |
| --- | --- | --- |
| Methods | Prospective, randomized, controlled, split-face study | |
| Participants | - Patients with erythematotelangiectatic rosacea - Total n = 16; female n = 8; 2 dropped out due to post-treatment swelling - mean age: 42 years - Fitzpatrick skin type: I (n = 1); II (n = 11); III (n = 2) | |
| Intervention | A: Nd:YAG laser  B: PDL | |
| Outcomes | - Follow-up: 4 weeks post-treatment - percent difference in spectrophotometer - preferred treated side - treatment satisfaction using a questionnaire - Pain intensity | |
| Notes | Parameters:   - 4 sessions at 3-4 intervals - 1064 nm Nd:YAG laser: 6 J/cm^2^; 8 mm spot size; 0.3 ms pulse duration - PDL: 7.5 J/cm^2^; 10 mm spot size; 6 ms pulse duration; 30 milliseconds/20 ms dynamic cooling device (DCD); 1 pass with overlap of 15% | |
| **Risk of bias** | | |
| **Bias** | **Authors’ judgement** | **Support for judgement** |
| Random sequence generation (selection bias) | Low risk | random number generator |
| Allocation concealment (selection bias) | Low risk | Each assignment was sealed in an opaque, sequentially numbered envelope. |
| Blinding of participants and personnel (performance bias) | Low risk | Subjects were blinded. First investigator was not blinded due to nature of treatment. |
| Blinding of outcome assessment (detection bias) | Low risk | Subjects and investigators for spectroscopic analysis were blinded. |
| Incomplete outcome data (attrition bias) | Low risk | 2 patients dropped out due to side effects. |
| Selective reporting (reporting bias) | Low risk | We judged this as low risk of bias. |
| Other bias | Low risk | The study appeared to be free of other forms of bias. |

| **Campos 2019** | | |
| --- | --- | --- |
| Methods | Prospective, randomized, controlled, double-blinded, split-face study | |
| Participants | - Patients with erythematotelangiectatic rosacea - Total n = 29; female n = 17; 2 dropped out due to post-treatment purpura or pain during treatment - mean age: 52.9 years - Fitzpatrick skin type: no details provided | |
| Intervention | A: PDL  B: PDL + Nd:YAG laser | |
| Outcomes | - Follow-up: before each treatment sessions, 1 month post-treatment - Spectrophotometer - Pain during treatment (0-10 scale) - Side effects - Purpura (0-100%) - treatment satisfaction (0-100%) - recommendation of the treatment to a friend | |
| Notes | Parameters:   - 3 sessions at 3-4 intervals - PDL: 6.0 J/cm^2^; 7 mm spot size; 0.5 ms pulse duration; level 3 of 5 DCD; 1 pass with overlap of 10% - PDL/ 1064 nm Nd:YAG: 7.0 J/cm^2^ for PDL and 35 J/cm^2^ for Nd:YAG; 7 mm spot size; 10 ms pulse duration for PDL and 15 ms for Nd:YAG; level 3 of 5 DCD; 1 pass with minimal overlap | |
| **Risk of bias** | | |
| **Bias** | **Authors’ judgement** | **Support for judgement** |
| Random sequence generation (selection bias) | Low risk | random number generator |
| Allocation concealment (selection bias) | Low risk | Each assignment was sealed in an opaque, sequentially numbered envelope. |
| Blinding of participants and personnel (performance bias) | Low risk | Subjects were blinded. First investigator was not blinded due to nature of treatment. |
| Blinding of outcome assessment (detection bias) | Low risk | Subjects and investigators for spectroscopic analysis were blinded. |
| Incomplete outcome data (attrition bias) | Low risk | 2 dropped out due to post-treatment purpura or pain during treatment |
| Selective reporting (reporting bias) | Low risk | We judged this as low risk of bias. |
| Other bias | Low risk | The study appeared to be free of other forms of bias. |

| **Handler 2017** | | |
| --- | --- | --- |
| Methods | Prospective, randomized, split-side study | |
| Participants | - Patients with facial erythema - Total n = 15; female n = 11; 13 were available at day 30 treatment; 14 were available at day 90 evaluation - Mean age: 53.9 years - Fitzpatrick skin type: I – II; no details provided | |
| Intervention | A: IPL  B: PDL | |
| Outcomes | - Follow-up: 90 days post-treatment - Change in erythema on a 5-point scale - Adverse events | |
| Notes | Parameters:   - 2 sessions at 30 days interval - IPL: 20 J/cm^2^; 30 ms; 20 °C - 595 nm PDL: 7 J/cm^2^; 30 ms; 20 °C | |
| **Risk of bias** | | |
| **Bias** | **Authors’ judgement** | **Support for judgement** |
| Random sequence generation (selection bias) | Unclear risk | No information provided. |
| Allocation concealment (selection bias) | Unclear risk | No information provided. |
| Blinding of participants and personnel (performance bias) | Unclear risk | No information provided. |
| Blinding of outcome assessment (detection bias) | Unclear risk | No information provided. |
| Incomplete outcome data (attrition bias) | Low risk | We judged this as low risk of bias. |
| Selective reporting (reporting bias) | Low risk | We judged this as low risk of bias. |
| Other bias | Low risk | The study appeared to be free of other forms of bias. |

| **Iyer 2006** | | |
| --- | --- | --- |
| Methods | Prospective, randomized, split-side study | |
| Participants | - Patients with facial erythema and telangiectasia - Total n = 9; female n = 6 - Mean age: no further details provided - Fitzpatrick skin type: I – II; no further details provided | |
| Intervention | A: PDL with purpuric reaction  B: PDL with subpurpuric reaction | |
| Outcomes | - Follow-up: 3 weeks post-treatment - Erythema on a 4-point scale - Vessel diameter on a 4-point scale - Density of telangiectasias - Vessel arborization on a 5-point scale | |
| Notes | Parameters:   - PDL with purpuric reaction: 12 J/cm^2^; 7 mm spot size; 6 ms pulse width; 20 ms cryogen cooling spray with a 10 ms delay; 1 pass with no overlap - PDL with subpurpuric reaction: 6 J/cm^2^; 10 mm spot size; 6 ms pulse width; 20 ms cryogen cooling spray with a 10 ms delay; 4 passes | |
| **Risk of bias** | | |
| **Bias** | **Authors’ judgement** | **Support for judgement** |
| Random sequence generation (selection bias) | Unclear risk | No information provided. |
| Allocation concealment (selection bias) | Unclear risk | No information provided. |
| Blinding of participants and personnel (performance bias) | Unclear risk | No information provided. |
| Blinding of outcome assessment (detection bias) | Unclear risk | No information provided. |
| Incomplete outcome data (attrition bias) | High risk | No information on baseline characteristics of patients were provided. |
| Selective reporting (reporting bias) | Low risk | We judged this as low risk of bias. |
| Other bias | High risk | Low sample size (n = 9). |

| **Karppinen 2019** | | |
| --- | --- | --- |
| Methods | Prospective, randomized, double blinded, split-side study | |
| Participants | - Patients with symmetrical facial telangiectasias - Total n = 24; female n = 16; 6 were excluded from analysis since their telangiectasias were not assessable with the imaging system - mean age: 48 years - Fitzpatrick skin type: I (n = 6); II (n = 8); III (n = 4) | |
| Intervention | A: KTP laser  B: yellow laser | |
| Outcomes | - Follow-up: 48-72 hours for adverse events, 1-2 months post-treatment - Telangiectasias on a 7-point scale - Pain intensity on a 0-100-point scale - Erythema, crusting, purpura, blisters on a 4-point scale | |
| Notes | Parameters:   - 1-2 sessions at 1-2 months interval - 532 nm KTP laser: 20 – 30 J/cm^2^; 1.0 mm spot size; 10 ms pulse duration - 585 nm yellow laser: 5.6 – 8.1 J/cm^2^; 1.4 mm spot size; 25 ms pulse duration | |
| **Risk of bias** | | |
| **Bias** | **Authors’ judgement** | **Support for judgement** |
| Random sequence generation (selection bias) | Low risk | A web-based validated program (Research Randomizer) was used. |
| Allocation concealment (selection bias) | Unclear risk | No further information was provided. |
| Blinding of participants and personnel (performance bias) | Low risk | Subjects were blinded. First investigator was not blinded due to nature of treatment. |
| Blinding of outcome assessment (detection bias) | Unclear risk | Investigators who conducted the treatments also assessed the outcome. While the assessment images did not reveal information about the laser used on each side, making the study appear blinded, there is still a potential risk of investigators recognizing their own patients. |
| Incomplete outcome data (attrition bias) | Low risk | We judged this as low risk of bias. |
| Selective reporting (reporting bias) | Low risk | We judged this as low risk of bias. |
| Other bias | Low risk | We judged this as low risk of bias. |

| **Karsai 2008** | | |
| --- | --- | --- |
| Methods | Prospective, randomized, controlled, single blinded, split-side study | |
| Participants | - Patients with pronounced nasal alar telangiectasia - Total n = 24; female n = 6 - Mean age: 62.4 ± 12.3 years - Fitzpatrick skin type: I – III; no further information provided | |
| Intervention | A: PDL + Nd:YAG laser  B: PDL  C: Nd:YAG laser | |
| Outcomes | - Follow-up: 4 weeks post-treatment - Clearance on a 4-point scale | |
| Notes | Parameters:   - 4 weeks post-treatment - 595 nm PDL: 10 J/cm^2^; 7 mm spot size; 10 ms pulse duration - Nd:YAG laser: multiplex interpulse delay of 100 ms; 70 J/cm^2^; 50 ms pulse duration - Cooling: air cooling level 4 during treatment, ice packs after treatment | |
| **Risk of bias** | | |
| **Bias** | **Authors’ judgement** | **Support for judgement** |
| Random sequence generation (selection bias) | Unclear risk | No further information on randomization process was provided. |
| Allocation concealment (selection bias) | Unclear risk | No further information was provided. |
| Blinding of participants and personnel (performance bias) | Unclear risk | No further information was provided. |
| Blinding of outcome assessment (detection bias) | Low risk | Three independent investigators assessed outcome. |
| Incomplete outcome data (attrition bias) | Low risk | We judged this as low risk of bias. |
| Selective reporting (reporting bias) | Low risk | We judged this as low risk of bias. |
| Other bias | Low risk | We judged this as low risk of bias. |

| **Kim 2011** | | |
| --- | --- | --- |
| Methods | Prospective, randomized, open, split‐side study | |
| Participants | - Patients with erythematotelangiectatic or papulopustular rosacea - Total n = 18; female n = 13; 3 withdrawals due to difficulty to attend follow-up visits - median age: 49; range 29 – 67 - Fitzpatrick skin type: III – V with 11 (62 %) having skin type IV | |
| Intervention | A: PDL + niacin  B: PDL | |
| Outcomes | - Follow-up: 6 weeks post-treatment - Improvement in erythema using a 4-point scale - Patient satisfaction using a 10-point scale | |
| Notes | Parameters:   - 3 sessions at 3 weeks interval - Thin layer of niacin cream was applied, then washed off after 20 minutes with 0.9 % saline solution - PDL: 7-9 J/cm2; 7 mm spot size; 10 ms pulse duration; 10 % overlap; air cooling level 3-4; ice packs post-treatment | |
| **Risk of bias** | | |
| **Bias** | **Authors’ judgement** | **Support for judgement** |
| Random sequence generation (selection bias) | Unclear risk | No further information was provided. |
| Allocation concealment (selection bias) | High risk | Randomization was not concealed from physicians who performed the treatments. |
| Blinding of participants and personnel (performance bias) | High risk | Subjects and investigators were not blinded due to the nature of study design. |
| Blinding of outcome assessment (detection bias) | Low risk | Three blinded dermatologists assessed outcome. |
| Incomplete outcome data (attrition bias) | Low risk | We judged this as low risk of bias. |
| Selective reporting (reporting bias) | Low risk | We judged this as low risk of bias. |
| Other bias | Low risk | We judged this as low risk of bias. |

| **Kim 2017** | | |
| --- | --- | --- |
| Methods | Prospective, randomized, controlled, single-blinded, split-face study | |
| Participants | - Patients with erythematotelangiectatic or papulopustular rosacea - Total n = 30; female n = 19 - mean age: 43.4 years; range: 35–69 years - Fitzpatrick skin type: no further information provided | |
| Intervention | A: Radiofrequency  B: PDL | |
| Outcomes | - follow-up: week 4, 8, and 12; 4 weeks post-treatment - rosacea symptoms (flushing, nontransient erythema, papules and pustules, telangiectasia, burning or stinging, plaques, dry appearance, edema, ocular manifestations, peripheral locations, phymatous changes, global assessment) using a 4-point scale - chromameter - therapeutic improvement using a 4-point scale - patient satisfaction using a 4-point scale - side effects and pain intensity using a 10-point scale | |
| Notes | Parameters:   - 3 sessions at 4 weeks interval - PDL: 8-9 J/cm^2^; 7 mm spot size; 6 ms pulse duration; 30 ms cryogen cooling spray with 30 ms before the laser pulse - Radiofrequency: 2 cm^2^ tip; 80-120 J/cm^2^; 3 °C contact cooling; 3 passes | |
| **Risk of bias** | | |
| **Bias** | **Authors’ judgement** | **Support for judgement** |
| Random sequence generation (selection bias) | Unclear risk | No further information was provided. |
| Allocation concealment (selection bias) | High risk | Randomization was not concealed from patients and physicians who performed the treatments due to nature of study design. |
| Blinding of participants and personnel (performance bias) | High risk | No blinding from patients and physicians who performed the treatments due to nature of study design. |
| Blinding of outcome assessment (detection bias) | Low risk | Two blinded dermatologist assessed outcome. |
| Incomplete outcome data (attrition bias) | Low risk | We judged this as low risk of bias. |
| Selective reporting (reporting bias) | Low risk | We judged this as low risk of bias. |
| Other bias | Low risk | We judged this as low risk of bias. |

| **Kim 2018** | | |
| --- | --- | --- |
| Methods | Prospective, randomized, controlled, single-blinded, split-side study | |
| Participants | - Patients with rosacea - Total n = 9; female n = 8 - mean age: 40.33 ± 13.13 years; range: 20-59 - Fitzpatrick skin type: II - IV | |
| Intervention | A: short-pulsed IPL  B: PDL | |
| Outcomes | - Follow-up: week 3, 6, 9, 12, and 15; 3 weeks post-treatment - Investigator’s global assessment scale - Patient’s global assessment scale - Erythema, telangiectasia, papules/pustules, and patient satisfaction using a 5-point scale - Erythema and melanin index using the spectrophotometry - Side effects - Pain intensity using a 0-10 scale | |
| Notes | Parameters:   - 4 sessions at 3 weeks interval - 555 cut-off filter IPL: 8 J/cm^2^; 1.5 ms pulse duration - PDL: 8 J/cm^2^; 7 mm spot size, 1.5 ms pulse duration - Ice packs post-treatment | |
| **Risk of bias** | | |
| **Bias** | **Authors’ judgement** | **Support for judgement** |
| Random sequence generation (selection bias) | Unclear risk | No further information was provided. |
| Allocation concealment (selection bias) | Unclear risk | No further information was provided. |
| Blinding of participants and personnel (performance bias) | Unclear risk | No further information was provided. |
| Blinding of outcome assessment (detection bias) | Low risk | Blinded dermatologists assessed outcome. |
| Incomplete outcome data (attrition bias) | Low risk | We judged this as low risk of bias. |
| Selective reporting (reporting bias) | Low risk | We judged this as low risk of bias. |
| Other bias | High risk | Small sample size (n = 9). No information on conflict of interest was provided. |

| **Luo 2020** | | |
| --- | --- | --- |
| Methods | Prospective, randomized, controlled, split-side study | |
| Participants | - Patients with papulopustular rosacea - Total n = 260; female n = 214; in the IPL group 23 dropped out (17 loss of follow-up, 6 due to adverse events); in the control group 10 dropped out (6 loss of follow-up, 4 due to aggravation of skin lesions) - mean age: 40.20 ± 10.76 years - Fitzpatrick skin type: III – IV; no further details provided | |
| Intervention | A: IPL  B: no treatment | |
| Outcomes | - Follow-up: monthly intervals during the first 6 months of the follow-up, then every 6 months for 1.5 years - Telangiectasias on a 4-point scale - Total efficacy rate based on total score of rosacea symptoms (flushing, non-transient erythema, telangiectasia, burning or stinging, plaques, dry appearance and edema, physician's global assessment and patient's global assessment) before and after treatment - Recurrence rate | |
| Notes | Parameters:   - After anti-mite treatment: 3 sessions at 4 weeks interval - 540 nm IPL: 10-16 J/cm^2^; 1.5x4 cm^2^ spot size; 12 ms pulse duration; 10-15 pulse interval; no overlap - Ice packs post-treatment | |
| **Risk of bias** | | |
| **Bias** | **Authors’ judgement** | **Support for judgement** |
| Random sequence generation (selection bias) | Unclear risk | No further information was provided. |
| Allocation concealment (selection bias) | Unclear risk | No further information was provided. |
| Blinding of participants and personnel (performance bias) | High risk | No blinding performed due to the nature of the study design. |
| Blinding of outcome assessment (detection bias) | Unclear risk | No further information was provided. |
| Incomplete outcome data (attrition bias) | Low risk | We judged this as low risk of bias. |
| Selective reporting (reporting bias) | Low risk | We judged this as low risk of bias. |
| Other bias | Low risk | We judged this as low risk of bias. |

| **Maxwell 2010** | | |
| --- | --- | --- |
| Methods | Prospective, randomized, controlled, split-side study | |
| Participants | - Patients with type I rosacea - Total n = 14; female n = 10; 2 drop outs due to worsening of skin lesions - Mean age: 44 years, range 31-71 - Fitzpatrick skin type: | |
| Intervention | A: KTP laser  B: no treatment | |
| Outcomes | - Follow-up: 8 visits in total; 2 weeks post-treatment - Improvement of erythema and telangiectasia on a 5-point scale | |
| Notes | Parameters:   - 6 sessions at 2 weeks interval - 532 nm KTP laser: 17-20 J/cm2; 700 µm spot size, 25-30 ms pulse duration | |
| **Risk of bias** | | |
| **Bias** | **Authors’ judgement** | **Support for judgement** |
| Random sequence generation (selection bias) | Low risk | Role of the dice. |
| Allocation concealment (selection bias) | Unclear | No further information was provided. |
| Blinding of participants and personnel (performance bias) | High risk | Due to the nature of study design, patients and investigator were not blinded. |
| Blinding of outcome assessment (detection bias) | Low risk | Evaluators were blinded. |
| Incomplete outcome data (attrition bias) | High risk | Details in statistical analysis are missing. |
| Selective reporting (reporting bias) | Low risk | We judged this as low risk of bias. |
| Other bias | High risk | Small sample size (n = 14). |

| **Nam 2019** | | |
| --- | --- | --- |
| Methods | Prospective, randomized, controlled, split-face study | |
| Participants | - Patients with diffused facial erythema and telangiectasia - Total n = 20, female n = 15; 1 dropped out due to personal problems - mean age: 41.5 years; range 21-59 - Fitzpatrick skin type: no further information was provided | |
| Intervention | A: diode-pulsed fractional KTP laser  B: PDL | |
| Outcomes | - Follow-up: 4 weeks post-treatment - telangiectasias on a 10-point scale - erythema using colorimetry - subject self-assessment using a 6-point scale - patient satisfaction using a 6-point scale - pain intensity - adverse events | |
| Notes | Parameters:   - 3 sessions at 4 weeks interval - 532 nm KTP laser: 0.035 J/cm^2^ microfluence; 3.86 J/cm^2^ total fluence; 20 × 20 mm^2^ square spot size; 5 ms pulse duration; 3 passes; no overlap - PDL: 7.5 J/cm^2^; 10 mm spot size; 6 ms pulse duration; 1 pass; 15 % overlap - Ice pack post-treatment | |
| **Risk of bias** | | |
| **Bias** | **Authors’ judgement** | **Support for judgement** |
| Random sequence generation (selection bias) | Unclear risk | No further information was provided. |
| Allocation concealment (selection bias) | Unclear risk | No further information was provided. |
| Blinding of participants and personnel (performance bias) | Unclear risk | No further information was provided. |
| Blinding of outcome assessment (detection bias) | Low risk | Two independent dermatologists assessed outcome. Random codes were assigned to serial clinical photographs. |
| Incomplete outcome data (attrition bias) | Low risk | We judged this as low risk of bias. |
| Selective reporting (reporting bias) | Low risk | We judged this as low risk of bias. |
| Other bias | Low risk | We judged this as low risk of bias. |

| **Neuhaus 2009** | | |
| --- | --- | --- |
| Methods | Prospective, randomized, controlled, single blinded, split-side study | |
| Participants | - Patients with moderate erythematotelangiectatic rosacea - Total n = 29; female n = 20; 1 dropped out due to excessive swelling and reaction - Mean age: 45.7 ± 10.6 years - Fitzpatrick skin type: I (n= 8); II (n = 19); III (n = 2) | |
| Intervention | A: nonpurpuragenic PDL  B: IPL  C: no treatment | |
| Outcomes | - Follow-up: at each visit, 4 weeks post-treatment - reflectance spectrophotometer - erythema and telangiectasia using a 4-point scale - patients assessment of treatment outcome (erythema, flushing, dryness, burning, itching, swelling, burning, overall skin sensitivity), overall improvement ,and tolerability using visual analog scales - patients were asked whether they would undergo the treatment again | |
| Notes | Parameters:   - 3 sessions at 4 weeks interval - 560 nm IPL: 25 J/cm^2^; pulse train of 2.4 and 6.0 ms separated by a 15-ms delay - PDL: 7 J/cm^2^; 10 mm spot size; 6 ms pulse duration | |
| **Risk of bias** | | |
| **Bias** | **Authors’ judgement** | **Support for judgement** |
| Random sequence generation (selection bias) | Low risk | Random number generator. |
| Allocation concealment (selection bias) | High risk | No concealment was applied. |
| Blinding of participants and personnel (performance bias) | High risk | Patients and investigator performing the treatment were aware of allocation. |
| Blinding of outcome assessment (detection bias) | Low risk | A blinded investigator assessed outcome. |
| Incomplete outcome data (attrition bias) | High risk | p-values were provided, but further details were not provided. |
| Selective reporting (reporting bias) | Low risk | We judged this as low risk of bias. |
| Other bias | Low risk | We judged this as low risk of bias. |

| **Nymann 2010** | | |
| --- | --- | --- |
| Methods | Prospective, randomized, controlled, split-side study | |
| Participants | - Patients with rosacea-associated telangiectasia - Total n = 40; no further information on gender proportion; 1 died due to unrelated causes - mean age: 54 years; range 42-63 - Fitzpatrick skin type: I (n = 1); II (n = 19); III (n = 19) | |
| Intervention | A: PDL  B: IPL | |
| Outcomes | - Follow-up: 3 months post-treatment - Telangiectasias on a 5-point scale - Pain intensity on a 0-10 scale - Adverse effects | |
| Notes | Parameters:   - 3 sessions at 6 weeks interval - IPL:   - Thin superficial vessels: PR applicator; 8-16 J/cm^2^; 10 ms; 1 pass  - Thin deeper vessels: VL-2 applicator; 10-18 J/cm^2^; 10 ms; 1 pass  - Medium-sized, superficial vessels: 9-15 J/cm^2^; 14 ms; 1 pass  - Medium-sized, deeper vessels: VL-2 applicator; 11-18 J/cm^2^; 14 ms; 1 pass  - Thick vessels: VL-2 applicator; 12-20 J/cm^2^; 20 ms; 1 pass   - PDL:   - Thin superficial vessels: 7-9 J/cm^2^; 7 mm; 6 ms; 2-3 pass; DCD 30/10  - Thin deeper vessels: 6-7,5 J/cm^2^; 10 mm; 6 ms; 2-3 pass; DCD 30/10  - Medium-sized, superficial vessels: 8-10 J/cm^2^; 7 mm; 10 ms; 2-3 pass; DCD 30/10  - Medium-sized, deeper vessels: 7-9 J/cm^2^; 10 mm; 6 ms; 2-3 pass; DCD 30/10  - Thick vessels: 10-12 J/cm^2^; 7 mm; 20 ms; 2-3 pass; DCD 30/10 | |
| **Risk of bias** | | |
| **Bias** | **Authors’ judgement** | **Support for judgement** |
| Random sequence generation (selection bias) | Low risk | Patients drew lots. |
| Allocation concealment (selection bias) | Low risk | Randomized allocation was concealed in opaque sealed envelopes. |
| Blinding of participants and personnel (performance bias) | Unclear risk | Randomization was carried out using opaque envelopes, but no further information on blinding of patients or investigators who performed the treatment was provided. |
| Blinding of outcome assessment (detection bias) | Low risk | One blinded investigator evaluated outcome. |
| Incomplete outcome data (attrition bias) | Low risk | We judged this as low risk of bias. |
| Selective reporting (reporting bias) | Low risk | We judged this as low risk of bias. |
| Other bias | Low risk | We judged this as low risk of bias. |

| **Osman 2022** | | |
| --- | --- | --- |
| Methods | Prospective, randomized, controlled, split-side study | |
| Participants | - Patients with erythematotelangiectatic or papulopustular rosacea - Total n = 30; female n = 25 female - mean age: 38.97 years; range 16-73 - Fitzpatrick skin type: III (n = 26); IV (n = 4) | |
| Intervention | A: PDL + ivermectin  B: PDL | |
| Outcomes | - Follow-up: before each treatment session, 3 months post-treatment - Improvement on a 4-point scale - Rosacea severity: mild, moderate, severe - Patient satisfaction on a 4-point scale - dermoscopy | |
| Notes | Parameters:   - 4 sessions at 4 weeks interval - PDL: 5-6,5 J/cm2; 5-7 mm spot size; 0.45 ms pulse duration; overlap by 1 mm - Ivermectin 1% cream: once daily in a thin layer for 12 weeks | |
| **Risk of bias** | | |
| **Bias** | **Authors’ judgement** | **Support for judgement** |
| Random sequence generation (selection bias) | Unclear risk | No further information was provided. |
| Allocation concealment (selection bias) | Unclear risk | No further information was provided. |
| Blinding of participants and personnel (performance bias) | High risk | Due no nature of study design (without using vehicle as a control). |
| Blinding of outcome assessment (detection bias) | Low risk | Two blinded independent investigators evaluated outcome. |
| Incomplete outcome data (attrition bias) | High risk | p-values were provided, but further details were only partly not provided. |
| Selective reporting (reporting bias) | Low risk | We judged this as low risk of bias. |
| Other bias | Low risk | We judged this as low risk of bias. |

| **Park 2016** | | |
| --- | --- | --- |
| Methods | prospective, randomized, split-side study | |
| Participants | - Patients with mild to moderate rosacea - Total n = 21; female n = 20 - mean age: 42.9 ± 10.3 years - Fitzpatrick skin type: III or IV (no further details provided) | |
| Intervention | A: fractional microneedling radiofrequency (FMR)  B: no treatment | |
| Outcomes | - Follow-up: at each treatment session, week 4 and 8 post-treatment - Erythema index using dermaspectrometer - a* value using spectrophotometer - histologic analysis - pain intensity on a 0-10-point scale - patient satisfaction on a 0-10-point scale | |
| Notes | Parameters:   - Two sessions at 4 weeks interval - FMR: tip with 49 insulated needles; 5-7,5 Watt; 50-70 ms conduction time; 2 passes; slight overlap | |
| **Risk of bias** | | |
| **Bias** | **Authors’ judgement** | **Support for judgement** |
| Random sequence generation (selection bias) | Unclear risk | No further information was provided. |
| Allocation concealment (selection bias) | Unclear risk | No further information was provided. |
| Blinding of participants and personnel (performance bias) | High risk | Due to nature of study design no blinding of participants or personnel could be established. |
| Blinding of outcome assessment (detection bias) | Low risk | Two blinded independent investigators assessed outcome. |
| Incomplete outcome data (attrition bias) | Low risk | We judged this as low risk of bias. |
| Selective reporting (reporting bias) | Low risk | We judged this as low risk of bias. |
| Other bias | Low risk | We judged this as low risk of bias. |

| **Park 2022** | | |
| --- | --- | --- |
| Methods | Prospective, randomized, single blinded, split-side study | |
| Participants | - Patients with erythematotelangiectatic or papulopustular rosacea - Total n = 27; female n = 18/23; 4 dropped out due to personal reasons - mean age: 41.5 years; range 21-64 - Fitzpatrick skin type: no details provided | |
| Intervention | A: alexandrite laser + Nd:YAG laser  B: PDL | |
| Outcomes | - Follow-up: 1 and 3 months post-treatment - Erythema index using spectrophotometer - global aesthetic improvement scale - patient satisfaction on a 5-point scale - adverse events | |
| Notes | Parameters:   - 4 sessions at 4 weeks interval - 755 nm Alexandrite laser: 10 J/cm^2^; 10 mm spot size; 20 ms pulse duration - 1064 nm Nd:YAG laser: 4 J/cm^2^; 10 mm spot size; 0.3 ms pulse duration - PDL: 8J/cm^2^; 10 mm spot size; 10 ms pulse width; subpurpuric | |
| **Risk of bias** | | |
| **Bias** | **Authors’ judgement** | **Support for judgement** |
| Random sequence generation (selection bias) | Unclear risk | No further information was provided. |
| Allocation concealment (selection bias) | Unclear risk | No further information was provided. |
| Blinding of participants and personnel (performance bias) | High risk | Due to nature of study design no blinding of participants or personnel could be established. |
| Blinding of outcome assessment (detection bias) | Low risk | Two blinded investigators assessed outcome. |
| Incomplete outcome data (attrition bias) | Low risk | We judged this as low risk of bias. |
| Selective reporting (reporting bias) | Low risk | We judged this as low risk of bias. |
| Other bias | Unclear risk | No conflict of interest statement provided. |

| **Seo 2013** | | |
| --- | --- | --- |
| Methods | Prospective, randomized, comparative, single blinded, parallel group study | |
| Participants | - Patients with erythematotelangiectatic or papulopustular rosacea - Total n = 49; female n = 18; 12 dropped out: 11 lost to follow-up, 1 due to post-treatment worsening after PDL - mean age: LPAN: 49.9 ± 12.8 years; PDL: 48.6 ± 11.3 years - Fitzpatrick skin type: III (n = 2); IV (n = 30); V (n = 5) | |
| Intervention | A: long-pulsed alexandrite + Nd:YAG laser (LPAN)  B: PDL | |
| Outcomes | - Follow-up: 2 weeks and 6 months post-treatment - erythema index using a spectrophotometer - physician's global assessment using a 4-point scale - patient satisfaction using a 5-point scale - pain intensity using a 10-point visual analog scale - adverse effects | |
| Notes | Parameters:   - 4 sessions at 4 weeks interval - 755 nm Alexandrite laser: 30 J/cm^2^; 12 ms pulse duration; 10 mm spot size; 2 passes; at least 15 % overlap - 1064 nm Nd:YAG laser: 3.0 J/cm^2^; 0.5 ms pulse duration; 10 mm spot size; 6 passes - PDL: 7 J/cm^2^; 6 ms pulse duration; 10 mm spot size; 2 passes; at least 15 % overlap | |
| **Risk of bias** | | |
| **Bias** | **Authors’ judgement** | **Support for judgement** |
| Random sequence generation (selection bias) | Unclear risk | Each subject number was assigned to one laser before any subject was assigned with a subject number. As no further information on access of investigator to subject numbers was provided, we judged this as unclear risk. |
| Allocation concealment (selection bias) | Unclear risk | No further information was provided. |
| Blinding of participants and personnel (performance bias) | High risk | Subjects and investigators were not blinded due to the nature of study design. |
| Blinding of outcome assessment (detection bias) | Low risk | Two blinded dermatologists assessed outcome. |
| Incomplete outcome data (attrition bias) | Low risk | We judged this as low risk of bias. |
| Selective reporting (reporting bias) | Low risk | We judged this as low risk of bias. |
| Other bias | Unclear risk | No conflict of interest statement was provided. |

| **Sodha 2021** | | |
| --- | --- | --- |
| Methods | Prospective, randomized, controlled, parallel group study | |
| Participants | Patients with erythematotelangiectatic Rosacea  - Total n = 34; female n = 23; 4 dropped out (oxymethazoline + PDL: 1 withdrawal; oxymetazoline: 1 could not attend follow-up visit, 1 withdrew prior to 1-month follow-up, 1 experienced breakout with oxymetazoline cream) - Mean age: 45 ± 11 years; range 26-61 - Fitzpatrick skin type: I (n =1); II (n =19); III (n =8); IV (n =2) | |
| Intervention | A: oxymethazoline + PDL  B: oxymetazoline | |
| Outcomes | - Follow-up: at each treatment session, 1 week after first treatment session; 3 months after last treatment session - Investigator clinical erythema assessment using a 5-point scale - Subject self assessment using a 5-point scale - Investigator and Subject global aesthetic improvement using a 5-point scale - improvement in vessel size using a 5-point scale - Patient satisfaction using a 5-point scale - Pain intensity using a 0-10-point scale - Adverse events | |
| Notes | Parameters:   - PDL: 3 sessions at 1 month interval; varying fluences, spot sizes, and pulse duration; clinical endpoint: immediate vessel blanching or subtle bluish graying with subsequent erythema (nonpurpuragenic) - Contact cooling or DCD (30/20 ms spray / 20 ms delay) - 1 % oxymetazoline hydrochloride cream: once daily for 6 months | |
| **Risk of bias** | | |
| **Bias** | **Authors’ judgement** | **Support for judgement** |
| Random sequence generation (selection bias) | Unclear risk | No further information was provided. |
| Allocation concealment (selection bias) | Unclear risk | No further information was provided. |
| Blinding of participants and personnel (performance bias) | High risk | Subjects and investigators were not blinded due to the nature of study design. |
| Blinding of outcome assessment (detection bias) | Unclear risk | No further information was provided. |
| Incomplete outcome data (attrition bias) | Low risk | We judged this as low risk of bias. |
| Selective reporting (reporting bias) | Low risk | We judged this as low risk of bias. |
| Other bias | High risk | One author serves on the medical advisory board for the laser company of the laser system used in the study. |

| **Tanghetti 2011** | | |
| --- | --- | --- |
| Methods | Prospective, randomized, controlled, split-side study | |
| Participants | - Patients with facial telangiectasias - Total n = 16; female n = 14 - Mean age: 63 years; range 35-85 - Fitzpatrick skin type: no details provided | |
| Intervention | A: PDL  B: IPL | |
| Outcomes | - Follow-up: 48-96 hours and 1-2 months post-treatment for safety; 3 months post-treatment for efficacy - Skin reaction using a 4-point scale - Telangiectasia Grading Score (TGS) using a 7-point scale - Patient satisfaction - Patients were asked which treatment they would recommend to friends and family | |
| Notes | Parameters:   - 1-2 sessions at 1-2 months interval - PDL for smaller vessels: 8.1–8.5 J/cm^2^; 10 ms pulse duration; 10 mm spot size; air cooling level 4; ultrasound gel - PDL for larger vessels: 14.5 J/cm^2^; 40 ms pulse duration; 7 mm spot size; air cooling level 4; ultrasound gel - IPL for smaller vessels: 34–40 J/cm^2^; 10 ms pulse duration; 10 x 15 mm spot size; 2-3 passes; no pulse stacking; 5 °C contact cooling; optical coupling lotion - IPL for larger vessels: 55–70 J/cm^2^; 100 ms pulse duration; 10 x 15 mm spot size; 2-3 passes; no pulse stacking; 5 °C contact cooling; optical coupling lotion - Clinical endpoint or both systems: vessel disappearance or dark gray to black discoloration of vessels | |
| **Risk of bias** | | |
| **Bias** | **Authors’ judgement** | **Support for judgement** |
| Random sequence generation (selection bias) | Low risk | Randomization by coin flip. |
| Allocation concealment (selection bias) | Unclear risk | No further information was provided. |
| Blinding of participants and personnel (performance bias) | Unclear risk | No further information was provided. |
| Blinding of outcome assessment (detection bias) | Low risk | Three blinded reviewers assessed outcome. |
| Incomplete outcome data (attrition bias) | Low risk | We judged this as low risk of bias. |
| Selective reporting (reporting bias) | Low risk | We judged this as low risk of bias. |
| Other bias | High risk | Study was sponsored by the IPL system company. |

| **Tierney 2009** | | |
| --- | --- | --- |
| Methods | Prospective, randomized, controlled, split-side study | |
| Participants | - Patients with facial telangiectasias - Total n = 10; female n = 6 - Mean age: 56.4 ± 13.06; range 42-82 - Fitzpatrick skin type: no details provided | |
| Intervention | A: 532 nm diode laser  B: 940 nm diode laser | |
| Outcomes | - Follow-up: at each treatment session, 2 months post-treatment - Telangiectasias using a 10-point scale - Side effects using a 6-point scale | |
| Notes | Parameters:   - 2 sessions at 6 weeks interval - 532 nm diode laser: 15 J/cm^2^; 60 ms pulse duration; 1 mm spot size - 940 nm diode laser: 100 J/cm^2^; 21 ms pulse duration; 1 mm spot size - Cooling with ice for 5 minutes | |
| **Risk of bias** | | |
| **Bias** | **Authors’ judgement** | **Support for judgement** |
| Random sequence generation (selection bias) | Low risk | Randomization by coin flip. |
| Allocation concealment (selection bias) | Unclear risk | No further information was provided. |
| Blinding of participants and personnel (performance bias) | Unclear risk | No further information was provided. |
| Blinding of outcome assessment (detection bias) | Low risk | Two blinded physicians assessed outcome. |
| Incomplete outcome data (attrition bias) | Low risk | We judged this as low risk of bias. |
| Selective reporting (reporting bias) | Low risk | We judged this as low risk of bias. |
| Other bias | High risk | Small sample size (n = 10). No conflict of interest statement was provided. |

| **Uebelhoer 2007** | | |
| --- | --- | --- |
| Methods | Prospective, randomized, single blinded, split-side study | |
| Participants | - Patients with diffuse facial telangiectasias due to photoaging or rosacea - Total n = 15; female n = 7 - Mean age: 52.4 years; range: 35–70 - Fitzpatrick skin type: I (n = 6); II (n = 6); III (n = 2); IV (n = 1) | |
| Intervention | A: KTP laser  B: PDL | |
| Outcomes | - Follow-up: at each treatment session; 3 weeks post-treatment - Patients were asked to select the side with the greatest improvement - Patients were asked to select the treatment which was least uncomfortable and degree and amount of posttreatment side effects including redness, swelling, and crusting | |
| Notes | Parameters:   - 3 sessions at 3 weeks interval - PDL: 7.5 J/cm^2^; 10 ms pulse duration; 10 mm spot size; 1 pass; at least 13 % overlap; stack pulses on all pronounced ectatic individual vessels; up to 2 passes on pronounced telangiectatic erythema; DCD 30 ms spray/ 20 ms delay - KTP laser for most prominent vessels: 8-11 J/cm^2^; 18-20 ms pulse duration; 5 mm spot size - KTP laser for the entire affected area: 7 -10 J/cm^2^; 20-25 ms pulse duration; 10 mm spot size; 1 pass; 5-10 % overlap - Patients were assigned to either prednisone for 3 days or ice packs immediately after treatment for 20 minutes and for 20 minutes every 2 hours for the next 4 hours | |
| **Risk of bias** | | |
| **Bias** | **Authors’ judgement** | **Support for judgement** |
| Random sequence generation (selection bias) | Unclear risk | Each subject number was assigned to one laser before any subject was assigned with a subject number. As no further information on access of investigator to subject numbers was provided, we judged this as unclear risk. |
| Allocation concealment (selection bias) | Unclear risk | No further information was provided. |
| Blinding of participants and personnel (performance bias) | High risk | Patients and investigator performing the treatment were not blinded. |
| Blinding of outcome assessment (detection bias) | Low risk | One blinded investigator assessed outcome. |
| Incomplete outcome data (attrition bias) | High risk | Standard deviations or ranges and statistical tests were not provided. |
| Selective reporting (reporting bias) | Low risk | We judged this as low risk of bias. |
| Other bias | High risk | Study was funded by the laser company of the KTP laser. |

| **Üstüner 2008** | | |
| --- | --- | --- |
| Methods | Prospective, randomized, controlled, double blinded, split-side study | |
| Participants | - Patients with erythematotelangiectatic or papulopustular rosacea - Total n = 30; female n = 13; 2 dropped out due to adverse events - mean age: 38.64 ± 13.33; range 22-67 - Fitzpatrick skin type: II (n = 19); III (n = 9) | |
| Intervention | A: KTP laser  B: Nd:YAG laser | |
| Outcomes | - Follow-up: before and after each treatment session; 6 months post-treatment - Erythema using a 4-point scale - Telangiectasia using a 4-point scale - Telangiectasia thickness categorized into 4 different groups - Change in telangiectasia thickness - Rosacea severity score using a 4-point scale - Clinical improvement using a 4-point scale - Patient satisfaction using a 4-point scale - Pain intensity using a 10-point scale - Adverse events - Dermatological Life Quality Index (Turkish version) | |
| Notes | Parameters:   - 4 sessions at 1 month interval - 585 nm KTP laser: 1.5-2 J/cm^2^; 3 mm spot size; air cooling level 4; clinical endpoint: pale maroon-cherry bruise of vessels - 1064 nm Nd:YAG laser: 90-110 J/cm^2^; 15 ms pulse duration; 4 mm spot size; 5 % overlap; air cooling level 4; clinical endpoint: complete disappearance of vessels | |
| **Risk of bias** | | |
| **Bias** | **Authors’ judgement** | **Support for judgement** |
| Random sequence generation (selection bias) | Unclear risk | No further information was provided. |
| Allocation concealment (selection bias) | Unclear risk | No further information was provided. |
| Blinding of participants and personnel (performance bias) | Low risk | The study was double-blinded. |
| Blinding of outcome assessment (detection bias) | Low risk | The study was double-blinded. |
| Incomplete outcome data (attrition bias) | High risk | p-values were provided, but further details were partly not provided. |
| Selective reporting (reporting bias) | Low risk | We judged this as low risk of bias. |
| Other bias | Low risk | We judged this as low risk of bias. |

| **West 1998** | | |
| --- | --- | --- |
| Methods | Prospective, randomized, controlled, split-side study | |
| Participants | - Patients with facial or leg telangiectasia - Total n = 12 leg telangiectasia; total n = 8 facial telangiectasia; details on gender not provided; 1 withdrawal of a patient with leg telangiectasia due to intolerable pain - mean age: 40; range 23-69 - Fitzpatrick skin type: I-III (no further details provided) | |
| Intervention | A: PDL  B: KTP laser | |
| Outcomes | - Follow-up: week 4, 8, 12, and 24; 8 and 12 weeks post-treatment - Pain intensity on a 10-point scale - Clearance on a 5-point scale - Adverse events | |
| Notes | Parameters:   - 1-2 sessions at 8 weeks intervals - For facial telangiectasia: PDL; 15 J/cm^2^; 1.5 ms pulse duration; 2x7 mm spot size - For facial telangiectasia: 532 nm KTP laser; 15 J/cm^2^; 10 ms pulse duration; 1 mm spot size | |
| **Risk of bias** | | |
| **Bias** | **Authors’ judgement** | **Support for judgement** |
| Random sequence generation (selection bias) | Unclear risk | No further information was provided. |
| Allocation concealment (selection bias) | Unclear risk | No further information was provided. |
| Blinding of participants and personnel (performance bias) | Unclear risk | No further information was provided. |
| Blinding of outcome assessment (detection bias) | Low risk | A physician, nurse, and the patient assessed outcome in a blinded manner. |
| Incomplete outcome data (attrition bias) | High risk | Results of facial and leg telangiectasia were partly not differentiated. |
| Selective reporting (reporting bias) | Low risk | We judged this as low risk of bias. |
| Other bias | Low risk | We judged this as low risk of bias. |

| **Yang 2023** | | |
| --- | --- | --- |
| Methods | Prospective, randomized, controlled, single blinded, parallel group study | |
| Participants | - Patients with moderate to severe rosacea - Total n = 44; female n = 34; 3 withdrawals (ALA-PDT: 1 treatment unavailable due to Covid pandemic and 1 due to personal reasons; minocycline: 1 due to adverse event) - mean age: 34 years; range 28-45 - Fitzpatrick skin type: no details provided | |
| Intervention | A: 5-aminolevulinic acid photodynamic therapy (ALA-PDT)  B: minocycline | |
| Outcomes | - Follow-up: 12 and 24 weeks post-treatment - Lesion count - Rosacea-specific quality of life score - Investigator global assessment score - Clinical erythema assessment score - Demodex density using reflectance confocal microscopy - Recurrence rate - Adverse events | |
| Notes | Parameters:   - ALA-PDT: 3-5 sessions at 1-2 weeks interval; 5 % ALA for 30 minutes; 633 ± 10 mm diode red light; 30 mW/ cm^2^ for 1h - Minocycline: 100 mg daily for 8 weeks | |
| **Risk of bias** | | |
| **Bias** | **Authors’ judgement** | **Support for judgement** |
| Random sequence generation (selection bias) | Low risk | Random numbers generated by a software. |
| Allocation concealment (selection bias) | Unclear risk | No further information was provided. |
| Blinding of participants and personnel (performance bias) | Unclear risk | Due to nature of study design, no blinding of participants or personnel was possible. |
| Blinding of outcome assessment (detection bias) | Low risk | Three blinded physicians assessed outcome. |
| Incomplete outcome data (attrition bias) | Low risk | We judged this as low risk of bias. |
| Selective reporting (reporting bias) | Low risk | We judged this as low risk of bias. |
| Other bias | Low risk | We judged this as low risk of bias. |
