# Supplementary material for: Laser and energy‐based devices for treating rosacea ‐ a systematic review and network meta‐analysis
Source: J Dtsch Dermatol Ges. 2025 Nov 21;24(1):24–32. doi: 10.1111/ddg.15961 (PMC12800891; doi:10.1111/ddg.15961)
Supplement: Supplementary file 3 — Supplementary information [file DDG-24-24-s005.docx]

**Characteristics of excluded studies [ordered by study ID]**

| **Study** | **Reason for exclusion** |
| --- | --- |
| Bao 2022 | Not controlled |
| Bennardo 2022 | Not controlled |
| Bernstein 2008 | Not controlled |
| Bernstein 2018 | Not controlled |
| Bernstein 2022 | Not controlled |
| Bernstein 2023 | Not controlled |
| Clark 2002 | Not controlled |
| Fan 2018 | Not controlled, data of acne vulgaris and rosacea were not differentiated |
| Kim 2021 | Not controlled |
| Kwon 2018 | Not randomized |
| Liu 2014 | Not controlled |
| McCoy 1997 | Not controlled |
| Park 2023 | Not controlled |
| Salem 2013 | Not randomized |
| Sun 2018 | Not controlled |
| Wang 2021a | Not controlled |
| Wang 2021b | Not controlled |
| Wang 2022 | Retracted |
| Tirico 2020 | Small sample size (n = 5) |

**Characteristics of studies awaiting assessment [ordered by study ID]**

| **NCT00945373** | |
| --- | --- |
| Methods | Prospective, open-label, split-face study |
| Participants | Patients with erythematotelangestatic rosacea |
| Intervention | A: 2.5% calcium dobesilate and PDL  B: PDL |
| Outcomes | Investigator Global Assessment (IGA) scale |
| Notes | - |

| **NCT01631656** | |
| --- | --- |
| Methods | Prospective, open-label, split-face study |
| Participants | 15 patients with mild to moderate rosacea |
| Intervention | A: azelaic acid 15% gel and Nd.YAG laser  B: Nd:YAG laser monotherapy |
| Outcomes | - Investigator's Global Assessment - Adverse events |
| Notes | Last update posted on 2018-09-12, study completion was 2011-02, website accessed 2023-12. Not yet published. |

| **NCT02075671** | |
| --- | --- |
| Methods | Prospective, randomized, double-blind, parallel group study |
| Participants | Patients with papulopustular rosacea |
| Intervention | A: 5 - aminolevulinic acid photodynamic therapy  B: vehicle and photodynamic therapy  C: vehicle |
| Outcomes | - Improvement of the Inflammatory Lesions (Papules, Pustules, Nodules), Erythema, and Telangiectasia using the Investigator's Global Assessment (IGA) and Inflammatory Lesion Investigator's Global Assessment (ILIGA) - Clinical Erythema Assessment (CEA) Scale - Difference in Inflammatory Lesion Count (ILC) - Patient Overall Assessment (POA) Scale |
| Notes | - |

| **NCT02204254** | |
| --- | --- |
| Methods | Prospective, open-label, randomized Study |
| Participants | Rosacea with papulopustular rosacea |
| Intervention | A: radiofrequency  B: 100 mg doxycycline |
| Outcomes | - severity of rosacea on a scale of 0-3 (Physician Global Assessment) - inflammatory lesions count - patient satisfaction - density of demodex |
| Notes | - |

| **NCT02268474** | |
| --- | --- |
| Methods | Prospective, Randomized, Controlled Split-Face Study |
| Participants | Patients with erythematotelangiectatic or papulopustular rosacea |
| Intervention | A: 532 nm KTP laser  B: PDL |
| Outcomes | Degree of Improvement on a 4-point scale |
| Notes | - |

| **NCT03424304** | |
| --- | --- |
| Methods | Prospective, open-label, split-face study |
| Participants | Patients with rosacea, wrinkles, and scarring (no further details provided) |
| Intervention | A: Cutera excel V™ laser and the Micro-Lens Array attachment to the Genesis V hand piece  B: Cutera excel V™ laser Green Genesis V hand piece to the CoolView hand piece |
| Outcomes | Global Aesthetic Improvement Scale |
| Notes | - |

| **NCT05771298** | |
| --- | --- |
| Methods | Open-label, prospective, parallel group study |
| Participants | Patients with erythematotelangiectatic or papulopustular rosacea |
| Intervention | A: 532 nm KTP laser  B: PDL |
| Outcomes | - reduction of erythema on a 5-point scale - pain intensity using the Numeric rating Scale - swelling on a 6-point scale - purpura on a 6-point scale - patient satisfaction on a 6-point scale |
| Notes | - |

**Characteristics of ongoing studies [ordered by study ID]**

| **ChiCTR2000038936** | |
| --- | --- |
| Study name | The efficacy and safety of pulsed dye laser in the treatment of rosacea |
| Methods | Prospective, parallel group study |
| Participants | Patients with rosacea |
| Intervention | A: PDL  B: IPL |
| Outcomes | - clinician improvement assessment - patient global improvement - Dermatology quality of life - Transeipdermal water loss - Erythema index - Melanin index - Pain intensity on a Visual Analog Scale |
| Starting date | 2020-10-12 |
| Contact information | Shuxian Yan  12 Middle Wulumuqi Road, Jing'an District, Shanghai 200032, China |
| Note | - |

| **ChiCTR2100042816** | |
| --- | --- |
| Study name | Clinical observation of 595nm dye laser in the treatment of rosacea using different sizes of light spot |
| Methods | Prospective, parallel group study |
| Participants | Patients with rosacea |
| Intervention | A: PDL with 10 mm spot  B: PDL with 7 mm spot |
| Outcomes | - Erythema value |
| Starting date | 2021-03-01 |
| Contact information | Di Wu  120 Guanghua Street, Wuhua District, Yunnan, China |
| Note | - |

| **ChiCTR2200066451** | |
| --- | --- |
| Study name | The effect evaluation of three types laser treatment for rosacea randomized trial |
| Methods | Prospective, randomized, parallel group study |
| Participants | Patients with rosacea |
| Intervention | A: IPL  B: DPL  C: Elos |
| Outcomes | - EI |
| Starting date | 2022-12-11 |
| Contact information | Chao Yuan  6A Build , 1278 Baode Road, Jingan District, Shanghai 200443, China |
| Note | - |

| **ChiCTR2300074594** | |
| --- | --- |
| Study name | The efficacy and safety of minocycline combined with radiofrequency induction of compound lidocaine cream in the treatment of rosacea |
| Methods | Prospective, controlled, parallel group study |
| Participants | Patients with rosacea with facial symptoms, and itching, burning, tingling and swelling |
| Intervention | A: minocycline with radiofrequency induction of compound lidocaine cream  B: Radiofrequency induction of compound lidocaine cream  C: Minocycline with radiofrequency induction of physiological saline  D: Radiofrequency induction of physiological saline |
| Outcomes | - symptom relief rate - Investigator’s Global Assessment score - Dermatology life quality index - Rosacea life quality index - Dermoscopy - Red zone score of VISIA - Transepidermal Water Loss, Stratum Corneum Hydration |
| Starting date | 2023-08-14 |
| Contact information | Li Xie  West China Hospital, Sichuan University, Chengdu, China |
| Note | - |

| **ChiCTR2300076423** | |
| --- | --- |
| Study name | A non-inferior randomized controlled study on the efficacy and safety of daylight photodynamic therapy (DL-PDT) for papulopustular rosacea (PPR) |
| Methods | Prospective, randomized, controlled, parallel group study |
| Participants | Patients with papulopustular rosacea |
| Intervention | A: Daylight photodynamic therapy (DL-PDT)  B: Conventional photodynamic therapy (c-PDT)  C: oral minocycline hydrochlorid |
| Outcomes | - Investigator’s Global Assessment (IGA) - skin lesion scores - clinician’s Erythema Assessment (CEA) - Quality of life using the rosacea-specific quality of life questionnaire (RosaQoL) - Dermatoscope - reflectance confocal microscopy |
| Starting date | 2023-10-09 |
| Contact information | Jian Li  Gaotanyan Main Street 30, Shapingba District, Chongqing,China |
| Note | - |

| **NCT05360251** | |
| --- | --- |
| Study name | Pulsed dye laser and intense pulsed light configured with different wavelength bands in improving erythematotelangiectatic rosacea |
| Methods | Prospective, randomized, controlled, single-blind, parallel group study |
| Participants | Patients with erythematotelangiectatic rosacea |
| Intervention | A: PDL  B: IPL (Delicate Pulsed Light)  C: IPL (M22 590)  D: IPL (M22 vascular filter) |
| Outcomes | - symptoms of rosacea (flushing, nontransient erythema, telangiectasia, burning or stinging, itching, dry) using a 4-point scale - VISIA Red Area score - Pain intensity on a Visual analogue scale(VAS) - Adverse events |
| Starting date | 2022-03-27 |
| Contact information | Suiqing S Cai  The Second Affiliated Hospital of Zhejiang University of Medicine, China |
| Note | - |

| **NCT05401422** | |
| --- | --- |
| Study name | Brimonidine in rosacea |
| Methods | Prospective, randomized, controlled, parallel group |
| Participants | Patients with erythematotelangiectatic rosacea |
| Intervention | A: doxycycline  B: doxycycline and PDL  C: doxycycline and brimonidine |
| Outcomes | - Clinical erythema assessment scale - Thickness of capillaries by dermoscope |
| Starting date | 2022-05-25 |
| Contact information | Hagar El Sayed  Cairo University |
| Note | - |

| **NCT06033352** | |
| --- | --- |
| Study name | Potassium-titanyl phosphate (KTP) laser vs KTP laser and ivermectin cream for facial rosacea (KIR) |
| Methods | Prospective, randomized, controlled, split-side study |
| Participants | Patients with facial erythematous rosacea or mild papulopustular rosacea with permanent erythema |
| Intervention | A: KTP laser  B: KTP laser and ivermectin cream |
| Outcomes | - assessment of erythema using the Normalized Erythema Index (NEI), Skin Redness Index (SRI), Clinical Erythema Assessment (CEA) - change in skin lesions using a 6-point Physician Global Assessment (PGA) - patient satisfaction using a 9-point visual analogue scale (VAS) - assessment of telangiectasia using a 4-point scale - change in the number of papules and papulopustules - assessment of swelling, redness, purpura - incidence of side effects and adverse events |
| Starting date | 2023-09-12 |
| Contact information | Kristine Heidemeyer, MD  Department of Dermatology, University Hospital Inselspital, Bern, Switzerland |
| Note | - |

| **NTR4804** | |
| --- | --- |
| Study name | Rosacea and the Subpurpuric pulsed dye laser treatment Efficacy |
| Methods | Prospective, Randomized, controlled, single-blinded, parallel group study |
| Participants | Patients with erythematotelangiectatic rosacea with at least 5 telangiectasia |
| Intervention | A: subpurpuric PDL with a maximum of 4 treatment at 2-weeks interval  B: subpurpuric PDL with a maximum of 4 treatment at 8-weeks interval |
| Outcomes | - Quality of life using the rosacea-specific quality of life questionnaire (RosaQoL) - evaluation of photographs using the Investigators Global Assessment (IGA) and Clinician's Erythema Assessment (CEA) - severity of telangiectasia using the Patient’s Global Assessment (PGA) |
| Starting date | 2013-03-01 |
| Contact information | van der Linden, M.M.D.  Department of Dermatology Academic Medical Center Meibergdreef 9, 1100 DD Amsterdam, The Netherlands |
| Note | - |
