# Supplementary figures and images for: Laser and energy‐based devices for treating rosacea ‐ a systematic review and network meta‐analysis
Source: J Dtsch Dermatol Ges. 2025 Nov 21;24(1):24–32. doi: 10.1111/ddg.15961 (PMC12800891; doi:10.1111/ddg.15961)

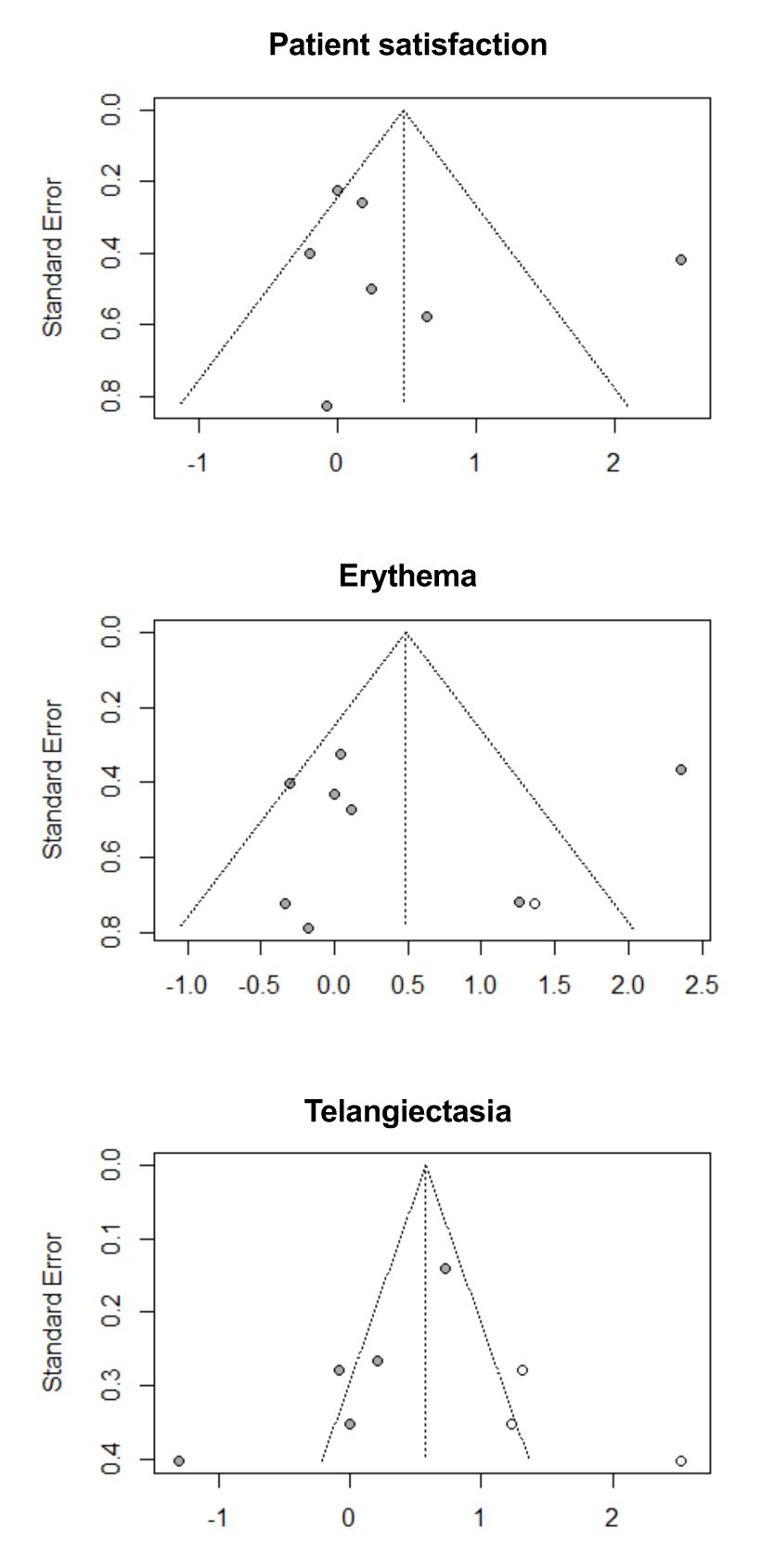

Supplement: Supplementary file 4 — Supplementary information [file DDG-24-24-s001.tif]

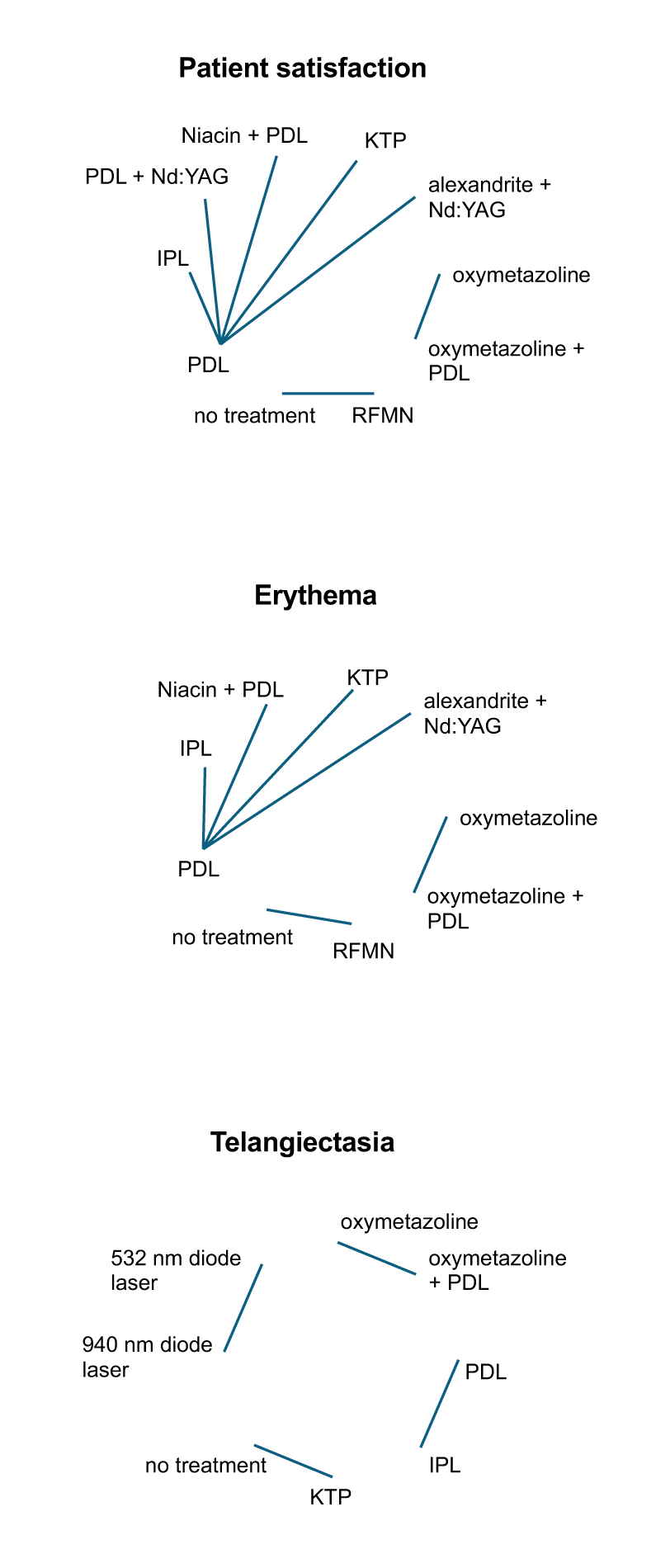

Supplement: Supplementary file 5 — Supplementary information [file DDG-24-24-s003.tif]
